# American Edition of Nothnagel’s Practice

**Published:** 1903-07

**Authors:** 


American Edition of Nothnagel's Practice.—Diseases of the Stomach. By Dr. F. Riegel, of Giessen. Edited, with additions, by Charles G. Stockton, M. D., Professor of Medicine in the University of Buffalo. Handsome octavo volume of 835 pages, illustrated, including 6 full-page plates. Philadelphia, New York, London: W. B. Saunders & Company. 1903. Cloth, $5.00 net; half Morocco, $6.00 net.

This volume, like the others of this excellent practice, is thorough and complete, scientific and practical. The importance of examining the stomach contents is becoming more and more apparent to the general practitioner. Methods in determining the presence or absence of hydrochloric acids, its bearing on the pathology of stomach disease, and the hydrochloric acid question in general is discussed with accuracy and clearness that spring from wide experience. The simplicity of the methods of chemical analysis of stomach contents and physical examination place this invaluable knowledge within easy reach of all, rendering treatment of the obscure conditions to be carried out in an intelligent and scientific manner. It is not necessary to be a chemist or to have a well-equipped laboratory to determine these necessary facts.

In this book we find particular attention given to disturbances of motility and their influence on the disturbances of secretion. It is evident that careful study has been devoted to the subject of impairment of the absorptive powers, and the significance of gas fermentation has been emphasized.

We are confident that for scientific excellence and completeness, as well as for mechanical perfection, this work stands unrivaled.

R. and L.

